# Digital health literacy and associated factors among internet users from China: a cross-sectional study

**DOI:** 10.1186/s12889-024-18324-0

**Published:** 2024-03-27

**Authors:** Bing-Yue Zhao, Long Huang, Xiao Cheng, Ting-Ting Chen, Si-Jia Li, Xiao-Juan Wang, Shui-Xiu Huang, Rong-Fang Hu, Hong Li

**Affiliations:** 1 School of Nursing, Fujian Medical University, 1 Xue Yuan Road, University Town, Fuzhou, 350122 China; 2 Department of Nursing, Zhuhai People’s Hospital (Zhuhai Clinical Medical College of Jinan University), Guangdong Zhuhai, China

**Keywords:** Digital health literacy, eHealth literacy, Internet use, Health-related information, Performance-based

## Abstract

**Background:**

As the internet develops and 5G technology becomes increasingly prominent, the internet has become a major source of health-related information. Increasingly, people use the internet to find health-related information, and digital health literacy is now a set of essential capabilities to improve their health in the digital era. However, little is known about the factors that influencing digital health literacy. This study aimed to assess digital health literacy scores and identify its influencing factors among internet users in China. Additionally, this study explored the participant’s actual skills using an additional set of performance-based items from the Digital Health Literacy Instrument (DHLI).

**Methods:**

An online cross-sectional study was conducted in August 2022. Participants aged ≥18 years were recruited to complete the survey. Data were collected using the Chinese revised version of the DHLI, the self-reported internet use questionnaire, and the sociodemographic questionnaire. We conducted multivariate linear regression analyses to explore the relationships among the sociodemographic variables, behavior of internet use, and the digital health literacy scores.

**Results:**

In total, 702 participants completed the survey. The mean DHLI score was 2.69 ± 0.61. Multivariate linear regression analyses showed that the age groups 35–49 (β = − 0.08, *P* = 0.033), 50–64 (β = − 0.161, *P* < 0.001), and ≥ 65 (β = − 0.138, *P* < 0.001) were negatively associated with DHL scores. However, education level, including bachelor’s or associate degree (β = 0.255, *P* = 0.002) and master’s degree and above (β = 0.256, *P* < 0.001), frequency of health-related Internet usage (β = 0.192, *P* < 0.001), the number of digital devices used (β = 0.129, *P* = 0.001), and OHISB (β = 0.103, *P* = 0.006) showed a positive relationship with DHL scores.

**Conclusions:**

The study findings demonstrate that age, educational levels, number of technological devices used, and greater use of the web for health information were independently associated with DHL scores. Healthcare providers should consider providing training programs tailored to specific sociodemographic factors to improve the ability that find and use accurate information online to meet digital health services, which contributes to enhance their self-management and reduce health disparities.

**Supplementary Information:**

The online version contains supplementary material available at 10.1186/s12889-024-18324-0.

## Background

Health literacy was recognized as a public health issue that plays a considerable role in improving health equity [1, 2]. Health literacy refers to the person’s ability to acquire, understand, and use information about health and health services [3]. Over the past several decades, people have acquired health information from magazines, best-selling books, television, and radio to improve their health. As the internet develops and 5G technology becomes increasingly prominent, sources for obtaining health information will shift from traditional media to mass digital media [4, 5]. Internet is a convenient way for the public to obtain health-related information, and people are more willing to seek health information onlin e[6]. As technology plays an increasing role, the skills required for health literacy have evolve d[7]. These skills are known as digital health literacy (DHL )[8] or eHealth literac y[9]. To better understand and apply health literacy in digital contexts and environments, Norman and Skinne r[9] proposed the definition of eHealth literacy in 2006. The concept of DHL developed from eHealth literacy and has since been refined. In the existing literature, DHL was often used interchangeably with eHealth literac y[10–12]. DHL refers to the ability to seek, find, understand, and appraise health information gathered from electronic sources and to apply the knowledge gained to solving a health problem. DHL shares core components of health literacy and encompasses additional skills. DHL as an essential literacy that combines six forms of literac y[9]: (1) traditional literacy, which encompasses basic literacy skills (e.g., the ability to read texts and understand written articles )[13]; (2) health literacy, which involves the ability of a person to acquire, understand and use information about health and health service s[3]; (3) information literacy, which is the ability refer to recognize when information is needed and to locate, evaluate, and use effectively the needed informatio n[14]; (4) scientific literacy, which is the ability of the nature, aims, methods, application, limitations, and politics of creating knowledge in a systematic manne r[15]; (5) media literacy, which is defined by the Trent Think Tank on Media Literacy as “the ability to decode, analyze, evaluate, and produce communication in a variety of forms” [16], briefly, media literacy refers to the attitude and ability to understand, judge, and use the media product; and (6) computer literacy, which is the ability to solve problems using computer s[17].

**Fig. 1:**
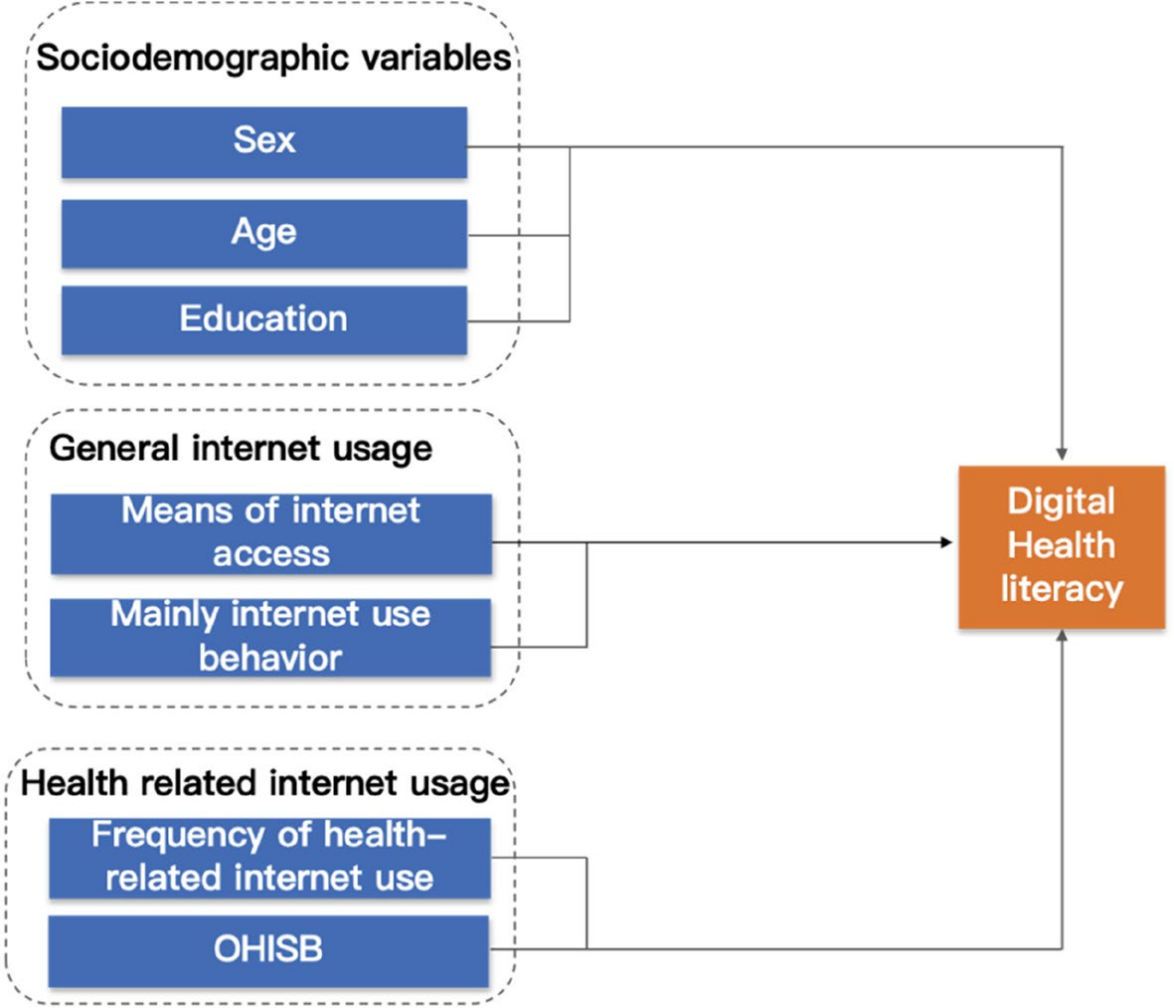
Conceptual framework in the present study. Note: OHISB=Online health information seeking behavior

According to internet World Stats, the number of internet users in China has increased from more than 22 million users in 2000 to over 1010 million in 2022, with a current internet penetration level of 69.8 %[18]. In 2018, the China State Council, together with the National Health and Wellness Commission, issued a series of documents that supporting healthcare institutions in building internet information platforms, including online health consultation, and health management services onlin e[19]. While the public may have access to more health information than ever, thanks to services such as the Patient Portal and Open Notes, accessing the vast amount of online information without adequate DHL skills can lead to confusion and stres s[7]. Additionally, the large amount of information generated by the internet, which may include false health information, can interfere with individuals’ abilities to make informed health decisions. Previous studie s[20–22] have shown that individuals with higher DHL scores may attempt to obtain health-related information that is as accurate as possible. Such individuals, also evaluate and use the information to maintain optimal self-caring health behaviors. There are significant differences in individuals’ DHL scores, online skills, and knowledge of the internet, which are related to individuals’ socioeconomic status and autonomy in using these tools [23, 24]. The Integrative Model of eHealth Use (IMeHU) proposed by Bodie and Dutta [25] provide a conceptual framework for understanding DHL and online health information adoption among internet users in the present study (see fig. 1). Internet use characteristics (e.g., whether a person owns or uses electronic devices), and personal characteristics, such as age, sex, and education, influence DHL.

### Sociodemographic determinants

IMeHU provides a conceptual framework for understanding how social structural (e.g., demographics) disparities influence on DHL. IMeHU suggests that the underlying social structure impacts an individual’s DHL level, and structural disparities contribute to healthcare disparities and overall well-being through DH L[25]. It is crucial to comprehend and analyze the correlation between DHL and sociodemographic factors, Such information may contribute to the development, implementation, and evaluation of digital interventions, ultimately aiding in preventing healthcare disparitie s[23, 26, 27]. In the existing studies [23, 28–30], the associated factors of DHL mainly include sex, age, educational background, income levels, marital status, etc. Previous studie s[31–33] have shown that younger people and individuals with higher educational levels tend to have greater DHL scores; they are more inclined to apply digital technology for health-related purposes. In addition, adults with lower educational levels may encounter comprehension barriers when searching for health information online [34]. Individuals with higher educational levels also tend to have higher DHL scores, allowing them to better access, understand and communicate operational online health information [35–37]. There is no consensus in the literature about sex differences regarding DHL. Hargittai et al. [38] found no significant difference in online information-seeking abilities between males and females. Additionally, previous studies [32, 39] found DHL scores did not differ by sex. However, a study [40] conducted among the general population in Hungary showed that males had lower DHL scores than females. In contrast, a study [41] including the health care workers showed that males had higher DHL scores than females, similar to the findings of previous studies conducted among college student s[42–44]. Furthermore, higher income levels have been associated with higher DHL scores [36, 37]. Additionally, a study [45] marital and occupational statuses also exhibit statistically significant differences in their impact on DHL scores, just as income levels do.

### Internet use

The popularity of the internet has made health information more availabl e[27]. People with higher internet usage and greater digital skills are more likely to be incentivized to adopt and use online health resources easil y[46]. Bach et al .[47] found that individuals who used digital health services frequently could better use health-related internet services for their decision-making process. In contrast, previous studies [48, 49] have found that content-related internet skills are not accompanied by an increase in internet experience or the frequency of online searching. The present study further explores the relationship between internet use and DHL.

Currently, information is lacking regarding the sociodemographic characteristics and internet use patterns associated with DHL among internet users in China. Therefore, this study conducted an online survey of internet users regarding their DHL scores. This study aimed to (a) determine the DHL levels of internet users in China, (b) analyze the participants’ actual skills using a set of performance-based items from the Digital Health Literacy Instrument (DHLI), and (c) explore associations between DHL levels and sociodemographic characteristics, internet use patterns.

## Methods

### Study design and participants

We conducted a web-based cross-sectional study using convenience and snowball sampling. Relevant data were collected by Fujian Medical University. The sample was recruited through several WeChat groups (nursing and related communities), including different occupations (e.g., medical staff, students, freelancers) who lived in different provinces of China (e.g., Henan, Fujian). The participants completed the online survey on the Questionnaire Star survey platform (www.wjx.cn )[50], which is a powerful, personalized platform for questionnaire design in Chin a[51]. The questionnaire indicated the study’s purpose, the respondents’ rights, and their rewards (each respondent had the opportunity to receive a WeChat red packet worth approximately RMB 2.88). Respondents were also encouraged to share the survey link with their peers. The inclusion criteria were as follows: (1) 18 years or older, (2) internet access, and (3) the ability to read Chinese. This study was approved by the Ethics Committee of Fujian Medical University (No: FMU2022093) and was conducted in accordance with the principles of the Helsinki Declaratio n[52]. As this study was voluntary and anonymous, no written informed consent was required, and participants were informed that clicking on the first page of the questionnaire was equivalent to giving their consent to participate and that they could stop the survey and withdraw from the study at any time.

### Measures

#### Digital health literacy

DHL was assessed using the Chinese revised version of DHLI (CR-DHLI )[53], originally developed by Van der Vaart and Drossaer t[10]. The DHLI contains 7 skill categories measured by 21 self-reported and 7 performance-based items. The scale measures the following skills: (1) operational skills, (2) navigation skills, (3) information searching, (4) evaluating reliability, (5) determining relevance, (6) adding content, and (7) protecting privacy. The self-reported items use a 4-point Likert scale, ranging from 4 (“very easy”) to 1 (“very difficult”) and from 4 (“never”) to 1 (“often”), so a higher score indicates a higher DHL score. For performance-based items, which asked participants to apply specific skills in fictional situations (Additional file 1), each item has 5 answer options: 4 different answers (of which 1 is correct) and an “I don’t know” option (score = 0). Each correct answer receives 1 point, adding up to a maximum total score of 7 points. To calculate a total score, at least 6 out of 7 items should be answered. The Cronbach’s α coefficient for the scale was 0.87.

We translated, revised, and culturally adapted of the DHLI for application in the Mandarin language. We applied the Brisli n[54] translation model, which includes forward translation (translation by two independent bilingual translators who majored in nursing), reverse translation (translation by two independent bilingual translators who majored in biomedical, English linguistics), and cultural adaptation (review by six experts who majored in health informatics, psychological education, and nursing). We then recruited 40 people to pre-test the translated version of the DHLI to ensure semantic idiomatic equivalence in Chinese characters for Mandarin speakers. Finally, the CR-DHLI was developed with a Cronbach’s α of 0.929 (Additional file 1). The self-report items use a 5-point Likert scale, ranging from 4 (“very easy”) to 0 (“very difficult”) and from 4 (“always”) to 0 (“never”), with higher scores indicating higher DHL scores. In the present study, the Cronbach’s alpha value of CR-DHLI was 0.931.

#### Sociodemographic characteristics and internet use

Demographic data collected for analysis included sex, age, educational levels, and occupation.

The self-developed survey questionnaire was designed based on the related literature [46, 55–58], including the following sections: (1) general internet usage: the means of internet access (i.e., mobile phone, computer/laptop, and tablet); the number of digital devices used (range 0–3); and main internet use behavior (i.e., watching movies/TV, watching short videos, online social, online shopping, online games, reading novels, information search, and study or work); (2) self-rated internet skills (excellent, good, fair, poor, or very poor); (3) online health information seeking behavior (OHISB): participants were asked whether they had used the internet to search for health-related information or advice in the last 3 months (yes/no); and (4) frequency of health-related internet use (frequency measured on a 5-point Likert scale ranging from 1 = “almost never” to 5 = “every day”);

### Data analysis

The data were analyzed using IBM SPSS Statistics 29.0 (IBM Corp. IBM SPSS Statistics for MAC, Version 29.0. Armonk, NY: IBM Corp.). This program was adopted to conduct descriptive and correlation analysis. Descriptive statistics were presented using means and standard deviation (SD) for parametric variables and absolute and relative frequencies for categorical variables. For normally distributed data, the intragroup differences were analyzed using the *t-*tests and analysis of variance (ANOVA). ANOVA and multiple comparisons (LSD) were conducted to compare the DHL scores of the participants with different demographic characteristics in subgroups. The Mann-Whitney test and Kruskal-Wallis tests were employed for non-parametric variables. Multivariate linear regression was performed to identify variables that were independently associated with the DHL scores (*P* < 0.05). In Model 1, DHL scores was considered the dependent variable, while significant sociodemographic factors (*P* < 0.05 in the univariate analysis) were considered the independent variables. In Model 2, the number of digital devices used, main internet use behaviors were analyzed as predictors. In Model 3, OHISB and frequency of health-related internet use were analyzed as predictors. Finally, Model 4 was adjusted to control for potential confounders (e.g., sex, age, and educational levels).

## Results

### Participant characteristics

A total of 800 internet users participated in the study. However, 98 invalid response questionnaires (12.3%) were excluded, representing an effective response rate of 87.75%. Table 1 shows the characteristics of the 702 included respondents. Slightly more than half of the participants were female (411/702, 58.5%). Of the participants, 76.6% (538/702) were younger than 35, over half of the participants had a bachelor or college degree, and 40.7% were students (286/702).

**Table 1:** Sociodemographic characteristics and comparisons of DHL scores among subgroups (*n* = 702)

Characteristics	n (%)	Statistics	***P***
**Sex**			
Male	291 (41.5)	−2.245^a^	**0.025**
Female	411 (58.5)		
**Age (years)**			
18–34	538 (76.6)	82.610^b^	**<0.001**
35–49	93 (13.2)		
50–64	56 (8.0)		
≥ 65	15 (2.1)		
**Educational level**			
Middle school and below	41 (5.8)	31.913^c^	**<0.001**
High School or technical secondary school	101 (14.4)		
Bachelor’s or associate degree	464 (66.1)		
Master’s degree and above	96 (13.7)		
**Occupation**			
Students	285 (40.7)	62.392^b^	**<0.001**
Health care professionals	126 (18.0)		
State agency workers	47 (6.7)		
Farmer/Worker	64 (9.1)		
Corporate/business staff	52 (7.4)		
Merchant/Service Staff	36 (5.1)		
Professional and technical staff	75 (10.7)		
Others	15 (2.1)		

^a^ Mann-Whitney Z test

^b^ Kruskal-Wallis H test

^c^ ANOVA

### Internet use patterns

Over half of the participants rated their internet skills as good and above (423/702, 60.3%) (Table 2*,* which placed at the end of the document text file). Over 90% of the participants accessed the internet through mobile phones, and over 70% reported that they frequently watched short-form videos online. Of the participants, 38.9% (273/702) reported accessing health-related internet content daily.

**Table 2:** Internet use patterns of participants (*n* = 702)

Characteristics	n (%)	Statistics	***P***
**Self-assessment of internet skills**			
Excellent	151 (21.5)	29.043^a^	**<0.001**
Good	272 (38.7)		
Fair	257 (36.6)		
Poor	22 (3.1)		
Very poor	0 (0)		
**Major digital devices used for internet access**			
Mobile phone	685 (97.6)	UA	UA
Computer or laptop	371 (52.8)		
Tablet	177 (25.2)		
**Major internet usage behaviors**			
Watching movies & TV	466 (66.4)	UA	UA
Watching short-form videos	524 (74.6)		
Online social	418 (59.5)		
Online shopping	443 (63.1)		
Online games	202 (28.8)		
Reading novels online	162 (23.1)		
Information searching	323 (46.0)		
Study/work	464 (66.1)		
**OHISB**			
whether the participant had looked for web-based health related information in the last 3 months			
No	448 (63.8)	−4.078^b^	**<0.001**
Yes	254 (36.2)		
**Frequency of health-related internet use**			
Every day	273 (38.9)	24.614^c^	**<0.001**
A few days a week	165 (23.5)		
About 1 day per week	109 (15.5)		
Less than 1 day per week	89 (12.7)		
Almost never	66 (9.4)		

^a^ ANOVA

^b^ Mann-Whitney Z test

^c^ Kruskal-Wallis H test

### Digital health literacy

**Fig. 2:**
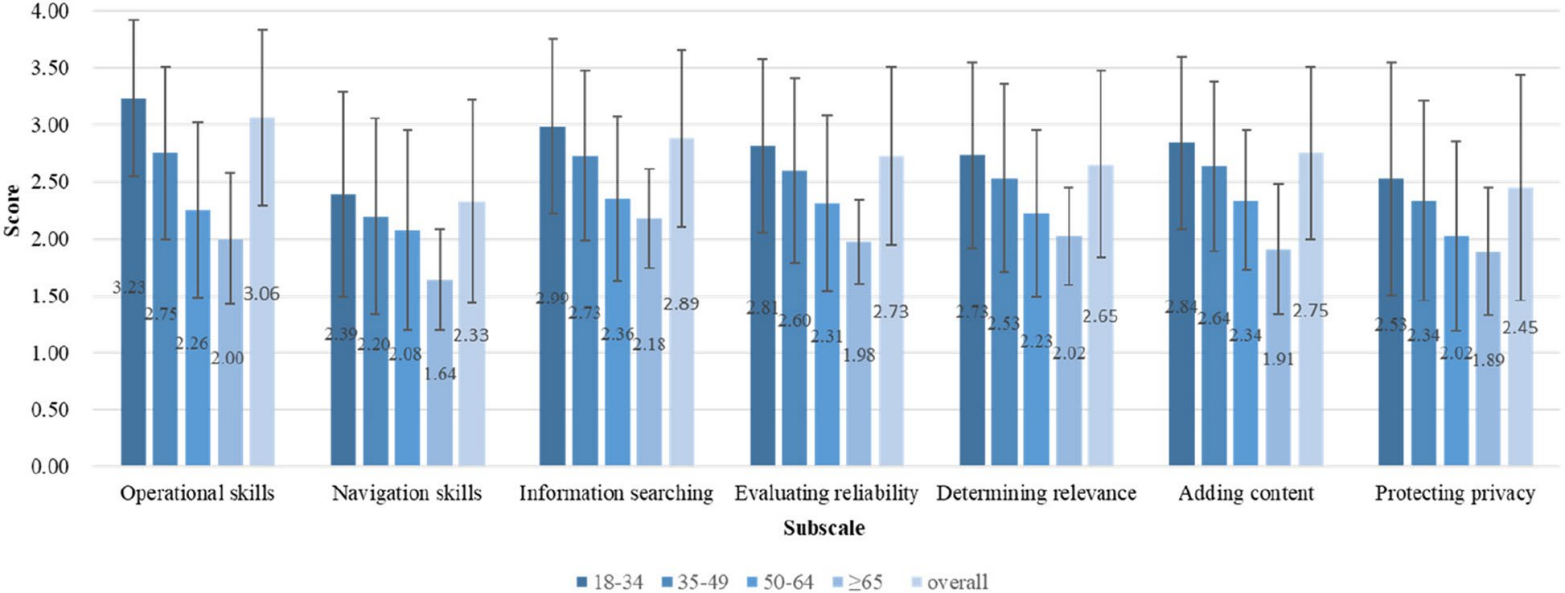
DHLI subscale mean scores for each age group and the entire study population (N = 702)

**Fig. 3:**
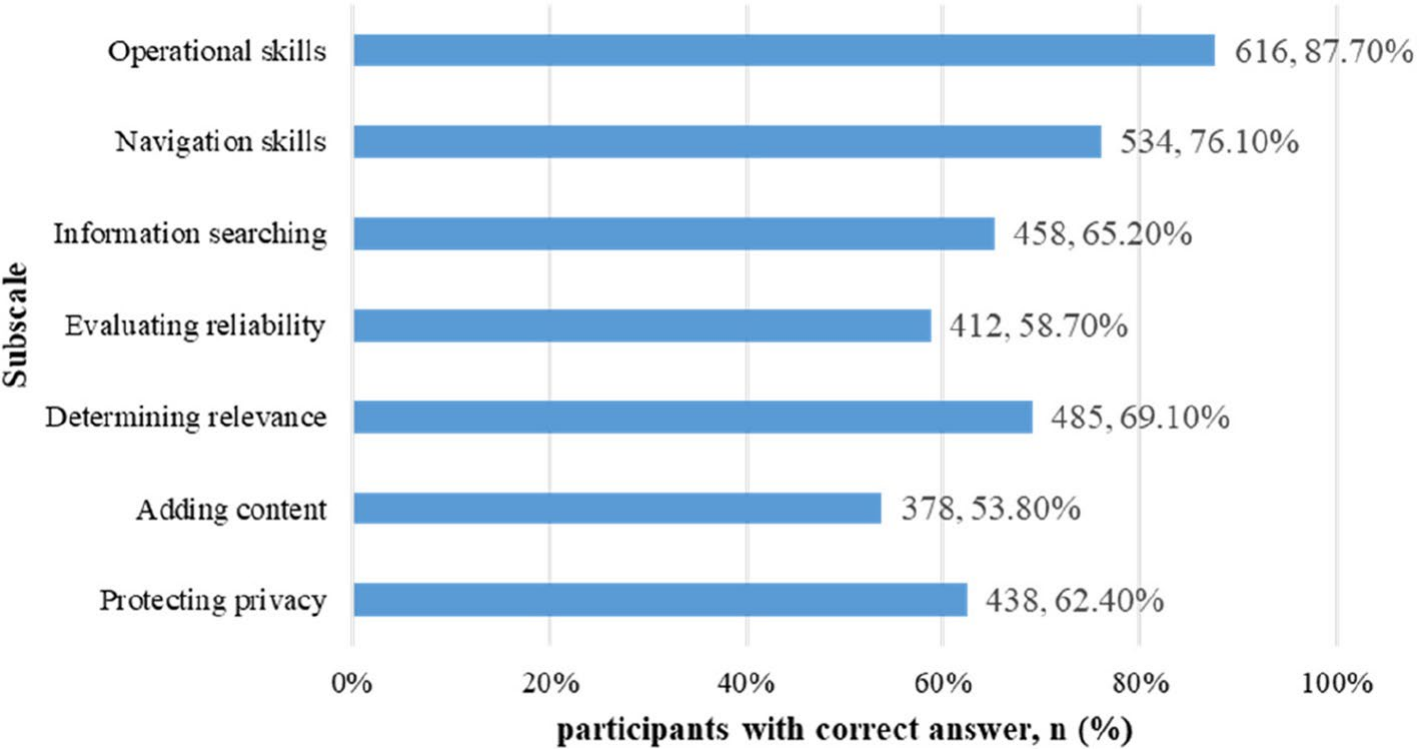
Number and percentages of the participants who answered the performance-based items correctly (n = 702)

Respondents had a total mean DHL score of 2.69 (SD = 0.61). The highest subscale score was reported for “operational skills” (mean = 3.06, SD = 0.77), while “navigation skills” had the lowest subscale score (mean = 2.33, SD = 0.89). The age group 18–34 scored higher on all subscales compared to the other age groups (see Fig. 2). Figure 3 describes the number of participants who answered each performance-based item correctly. Most respondents answered the items correctly. However, the subscale “adding content” had the largest proportion of respondents with an incorrect answer. Additional File 2 shows that the DHL scores were significantly higher among the respondents who self-rated their internet skills as good and above.

### Predictors of digital health literacy

Univariate analysis revealed that sex (*P* < 0.001), age (*P* < 0.001), and educational level (*P* < 0.001) were significantly associated with DHL (see Table 1). According to the final model (R^2^ = 0.294, Adjusted R^2^ = 0.276, F = 15.830, *P* < 0.001) (Table 3). The following five independent predictive variables exerted an influence on DHL: age, including 35–49 years (β = − 0.08, *P* = 0.033), 50–64 years (β = − 0.161, *P* < 0.001), and ≥ 65 years (β = − 0.138, *P* < 0.001) were negatively associated with DHL scores. However, education level, including bachelor’s or associate degree (β = 0.255, *P* = 0.002) and master’s degree and above (β = 0.256, *P* < 0.001), frequency of health-related internet usage (β = 0.192, *P* < 0.001), the number of digital devices used (β = 0.129, *P* = 0.001), and OHISB (β = 0.103, *P* = 0.006) showed a positive relationship with DHL scores. Multivariate linear regression analyses showed significant associations between OHISB and DHL scores after adjusting for sex, age, and level of education (*P* = 0.006). However, we found no association between main internet use behaviors of participants and the DHL scores (*P* > 0.05).

**Table 3:** Multiple linear regression analysis predicting DHL (N = 702)

Variable	Model 1			Model 2			Model 3			Model 4		
	Beta	*P*	VIF	Beta	*P*	VIF	Beta	*P*	VIF	Beta	*P*	VIF
Sex												
Male	Ref	–	–	–	–	–	–	–	–	Ref	–	–
Female	−0.034	0.351	1.07	–	–	–	–	–	–	− 0.03	0.414	1.24
Age (years)												
18–34	Ref	–	–	–	–	–	–	–	–	Ref	–	–
35–49	−0.086	**0.02**	1.106	–	–	–	–	–	–	−0.08	**0.033**	1.275
50–64	−0.147	**< 0.001**	1.375	–	–	–	–	–	–	−0.161	**< 0.001**	1.624
≥ 65	−0.141	**< 0.001**	1.149	–	–	–	–	–	–	−0.138	**< 0.001**	1.199
Educational level												
Middle school and below	Ref	–	–	–	–	–	–	–	–	Ref	–	–
High School or technical secondary school	0.116	0.062	3.126	–	–	–	–	–	–	0.111	0.063	3.251
Bachelor’s or associate degree	0.336	**< 0.001**	5.346	–	–	–	–	–	–	0.255	**0.002**	5.883
Master’s degree and above	0.35	**< 0.001**	3.573				–	–	–	0.256	**< 0.001**	4.105
The number of digital devices used (0–3)	–	–	–	0.233	**< 0.001**	1.298	–	–	–	0.129	**0.001**	1.466
Mainly activities on the Internet												
Watching movies & TV	–	–	–	0.078	0.042	1.188	–	–	–	0.049	0.183	1.232
Watching short-form videos	–	–	–	−0.053	0.153	1.092	–	–	–	0	0.99	1.146
Online social	–	–	–	0.047	0.259	1.407	–	–	–	−0.003	0.942	1.543
Online shopping	–	–	–	0.036	0.37	1.337	–	–	–	0.027	0.505	1.511
Online games	–	–	–	0.063	0.112	1.274	–	–	–	0.027	0.495	1.382
Reading novels online	–	–	–	0.047	0.222	1.182	–	–	–	0.055	0.129	1.215
Information searching	–	–	–	0.056	0.167	1.34	–	–	–	−0.002	0.964	1.438
Study/work	–	–	–	0.066	0.093	1.244	–	–	–	0.043	0.251	1.296
Average frequency of health-related Internet usage (1–5)	–	–	–	–	–	–	0.181	**< 0.001**	1.056	0.192	**< 0.001**	1.112
OHISB (whether the participant had looked for web-based health related information in the last 3 months)												
No	–	–	–	–	–	–	Ref	–	–	Ref	–	–
Yes	–	–	–	–	–	–	0.108	**0.004**	1.056	0.103	**0.006**	1.294

Bold: Statistically significant differences

## Discussion

This study examined the DHL scores of internet users from China and explored the impact of sociodemographic and personal factors on the DHL scores. The internet users in China exhibit a moderate DHL score. In addition, age, educational levels, the number of digital devices used, frequency of health-related internet use and OHISB were significant predictors of DHL. Furthermore, this study measured DHL with the CR-DHLI, which included 21 self-assessed items supplemented with 7 performance-based items to measure digital skills in a health context. This approach, which combines actual skills and self-assessments was intended to measure DHL scores more objectively. Our findings not only expand the research content in the DHL domain, but also provide a reference for the development of digital health services and the implementation of digital health interventions programs.

### Digital health literacy scores

This study’s participants reported feeling quite confident in their ability to use the internet. Over half of the participants reported adequate and better DHL skills. In the study, the participants mainly consisted of younger adults, and approximately 70% achieved tertiary education, which may explain why our results show similar findings to college student population from other studies [12, 59–61]. Among all the subscales examined, operational skills (basic skills for using the internet) displayed a high ceiling effect. Meanwhile, the participants were confident in their abilities to search and evaluate online health-related information. Additionally, the lowest subscales scores were reported on navigation skills self-report subscale. Navigation skills refer to whether an individual chooses to search online for certain health information, this finding could indicate that only a minority of the participants may believe they use the internet to seek and browse health-related information. When assessed through performance-based items within the navigation skills subscale, it was revealed that over 70% of participants resort to web searches for quick access to health information. Interestingly, the participants scored higher on the subscale adding content (self-reported), while attaining the highest proportion of incorrect answers on the corresponding performance-based item. These results are similar to previous study [10] conducted in Holland. Perhaps the reason is that people tend to overestimate or underestimate their internet skill s[62].

### Sociodemographic characteristics and digital health literacy

The DHL scores examined in this study varied by such personal characteristics as age, educational level, and sex. Age was negatively associated with DHL scores, which aligns with previous studie s[29, 46]. While internet use for seniors is becoming increasingly common, especially for those seniors who use the internet, the internet is a trusted source of health-related informatio n[63], compared with “digital natives” (i.e., those who have grown up in the digital era), Seniors still face more barriers and challenges in using technology for health information than do younger adults, due to the cognitive, motor, and sensory declines associated with agin g[48, 64–66]. In addition, educational scores are positively associated with DHL. Higher educational levels were associated with higher DHL scores. People with higher educational level had greater literacy and more confidence in their abilities to access and understand health-related information in a complex informational environmen t[46]. These results are consistent with the study by Neter and Braini n[32], that is, people with high DHL scores are younger and more educated.

Although previous studie s[40, 67–69] have reported significantly higher DHL scores for females. Our study analyzed the relationship between sex and the participants’ DHL scores, with no statistically significant results, which aligns with the finding of previous studie s[32, 46, 70]. This factor could be explained by the different social behaviors of males and females. Traditionally, males use the internet more actively and report higher digital skills scores than females d o[71]. However, previous studie s[47, 72] that have found that females (vs. males) are more sensitive to and interested in health information on social media. Women place a greater importance on healthy lifestyles than men d o[73], females are more likely to seek health-related information online owing to the role they play in their familie s[72, 74, 75], and women search for health information not only for themselves but also for their children or partner s[76, 77]. More research is needed to confirm the impact of sex on DHL.

### Internet use and digital health literacy

The contextual framewor k[25] in the present study suggests that to understand individual’s DHL, internet use characteristics should be considered in addition to demographic characteristics. We found that internet use patterns played an important role in improving DHL scores. Internet use patterns (e.g., the number of digital devices used, the frequency of health-related internet use) were associated with DHL, a finding that was in line with previous studie s[27, 78, 79], which indicated that DHL skills are influenced by information technology usage, and online health information seeking experience. The result aligns with a previous stud y[79] that showed a higher proportion of individuals with two or more electronic devices demonstrated high DHL scores compared to those with one or no electronic devices. Our findings also indicate that participants who actively sought health-related information or advice on the internet within the last three months (OHISB) exhibited higher DHL scores. In addition, the DHL scores increased along with the use of health-related internet services. Online health information seeking (OHIS) plays an important role in individuals’ health managemen t[80, 81]. However, among the surveyed internet users in this study, only 36.2% (254/702) were online health-related information seekers over 3 months. The abilities to navigate and comprehend online health information are often dependent on an individual’s DHL level, recognizing this link is crucial for crafting effective health communication strategies and digital tools, empowering individuals to make informed decisions about their health based on online information. Furthermore, the study found no significant correlation between primary internet usage behaviors and DHL.

The participants who perceived their skills as good tended to score mostly in the third and fourth quartile of the answer range, meaning that differences were small between their self-perceived digital health literacy and actual ability. A previous stud y[82] showed a gap between self-perceived skills and actual performance on web-based health-related assignments. People tend to overestimate or underestimate their own internet skills. An experimental study of online search behavior s[83] also found a gap between self-perceived efficacy in using online health information and actual use ability. However, the DHLI is a relatively objective assessment tool that strives to overcome this bias [10].

Our study lacked a thorough evaluation of the six domains of DHL, first, because such an assessment would require longer questionnaires that would take more time to complete, which might discourage participation [84]. Second, Norman [85] considered that DHL combining six different literacy skills. Focusing solely on one or two aspects of literacy within the model during assessments poses challenges when making claims about DHL as a whole, given that the concept is intended to represent a set of integrated skills. DHL operates within a learning system, where its component parts function collectively, rather than being easily amenable to subdivision.

### Limitation

This study has several limitations. First, it used a cross-sectional design, so causality cannot be established. Second, the sample size was unbalanced among the various age groups, and young adults in particular were overrepresented. Third, these similar studies used different scales, although we converted to comparable values. However, care must be taken when making comparisons, this situation also prompted us to consider these differences. Last, due to the inclusion of a student population in our sample, we opted not to collect data on marital status and income.

## Conclusion

The study findings demonstrate that age, educational levels, number of technological devices used, and greater use of the web for health information were independently associated with DHL scores. Digital health services are changing how individuals manage their health and participate in their care. The advancement of digital media and communication technology has expanded access to health-related information. Healthcare providers should consider providing training programs tailored to specific sociodemographic factors to improve the ability that find and use accurate information online. This situation was especially true for people who aging and lower educated, enabling them to effectively engage with digital health care services and tools for accessing relevant online health resources, which contributes to enhance their self-management and reduce health disparities.

### Supplementary Information

**Additional file 1.** Supplementary Material 1

## Data Availability

The datasets used during the current study are available from the corresponding author on reasonable request.

## Abbreviations

DHL: Digital Health Literacy
DHLI: Digital Health Literacy Instrument
OHISB: Online Health Information Seeking Behavior
