# Supplementary material for: Digital health literacy and associated factors among internet users from China: a cross-sectional study
Source: BMC Public Health. 2024 Mar 27;24:908. doi: 10.1186/s12889-024-18324-0 (PMC10976739; doi:10.1186/s12889-024-18324-0)
Supplement: Supplementary file 1 — Additional file 1. Supplementary Material 1 [file 12889_2024_18324_MOESM1_ESM.docx]

Digital Health Literacy and the Associated Factors among Internet Users from China: A Cross-Sectional Study

Additional file

Additional file 1 Chinese revised version-Digital Health Literacy Instrument (English translation version).

Additional file 2 Comparisons of digital health literacy scores among different self-assessed internet skills subgroups.

Additional file 1 Chinese revised version-Digital Health Literacy Instrument (English translation version).

| 1. How easy or difficult is it for you to… | Very  easy | Rather  easy | Moderate | Rather  difficult | Very  difficult |
| --- | --- | --- | --- | --- | --- |
| use the keyboard of a computer… | □ | □ | □ | □ | □ |
| use the web browser… | □ | □ | □ | □ | □ |
| use the buttons of seaching on websites… | □ | □ | □ | □ | □ |
| (performance-based items-1) When browsing websites using browsers on computers, and mobile devices, the function of the 'bookmark' is··· | | | | | |
| 2. When you search the internet for health information, how often does it happen that… | Never | Some-  times | Moderate | Often | Mostly |
| use the web to seek… | □ | □ | □ | □ | □ |
| participate in online discussions to… | □ | □ | □ | □ | □ |
| know where to get it online… | □ | □ | □ | □ | □ |
| (performance-based items-2) If you're seeking rapid access to health education information right now, what would be the most effective approach··· | | | | | |
| 3. When you search the internet for information on health, how easy or difficult is it for you to… | Very  easy | Rather  easy | Moderate | Rather  difficult | Very difficult |
| use a variety of online channels to… | □ | □ | □ | □ | □ |
| use the proper words or search query to… | □ | □ | □ | □ | □ |
| find the exact information you are looking for… | □ | □ | □ | □ | □ |
| (performance-based items-3) Imagine you are exploring the healthcare website below. You want to know what the website introductions are. Which button would you click on··· | | | | | |
| 4.When you search the internet for information on health, how easy or difficult is it for you to… | Very  easy | Rather  easy | Moderate | Rather  difficult | Very difficult |
| decide whether the information is suitable… | □ | □ | □ | □ | □ |
| make a choice from all the information … | □ | □ | □ | □ | □ |
| to use the information you found… | □ | □ | □ | □ | □ |
| (performance-based items-4) Imagine, you are receiveing treatment in the digestive diseases department in below hospital. You visit the department’s website. You browse around on the website. What kind of information do you expect to find when you click on that button··· | | | | | |
| 5. When you search the internet for information on health, how easy or difficult is it for you to… | Very  easy | Rather  easy | Moderate | Rather  difficult | Very difficult |
| decide whether the information is reliable… | □ | □ | □ | □ | □ |
| decide whether the information is written by… | □ | □ | □ | □ | □ |
| check different websites to see whether… | □ | □ | □ | □ | □ |
| (performance-based items-5) Imagine, You want to find out what the exact symptoms are of Varicose veins. A search via Google gives you the results below. Which of these results would most likely give you a correct and reliable answer··· | | | | | |
| 6. When typing a message, how easy or difficult is it for you to… | Very  easy | Rather easy | Moderate | Rather  difficult | Very difficult |
| clearly formulate your question… | □ | □ | □ | □ | □ |
| express your opinion, thoughts in writing… | □ | □ | □ | □ | □ |
| write your message as such, for people to… | □ | □ | □ | □ | □ |
| (performance-based items-6) Imagine, you recently enjoyed hotpot outside, had cold water during the meal, and subsequently experienced mild diarrhea upon returning home, and now choose to consult a doctor online, please share the details of your inquiry with the online doctor below··· | | | | | |
| 7. When you post a message on a public forum or social media, how often… | Never | Some-times | Moderate | Often | Mostly |
| do you find it difficult to… | □ | □ | □ | □ | □ |
| do you protect your own private information… | □ | □ | □ | □ | □ |
| remove web traces in a timely manner… | □ | □ | □ | □ | □ |
| (performance-based items-7) Here below you find three messages from patients, or their relatives, on a discussion forum. Which of the messages takes privacy into account properly··· | | | | | |

Additional file 2 Comparisons of digital health literacy mean scores among different self-assessed internet skills subgroups. (n=702)

| Self-assessed internet skills | N (%) | Mean | SD | *F* value | *P* |
| --- | --- | --- | --- | --- | --- |
| Excellent | 151 (21.6) | 3.01 | 0.64 | 41.256 | <0 .001 |
| Good | 272 (3 8.7) | 2.77 | 0.52 |  |  |
| Fair | 257 (36.6) | 2.49 | 0.55 |  |  |
| Poor | 22 (3.1) | 1.94 | 0.60 |  |  |
| Very poor | 0 (0) | --- | --- |  |  |
